# Classification of MR-Detected Additional Lesions in Patients With Breast Cancer Using a Combination of Radiomics Analysis and Machine Learning

**DOI:** 10.3389/fonc.2021.744460

**Published:** 2021-12-02

**Authors:** Hyo-jae Lee, Anh-Tien Nguyen, So Yeon Ki, Jong Eun Lee, Luu-Ngoc Do, Min Ho Park, Ji Shin Lee, Hye Jung Kim, Ilwoo Park, Hyo Soon Lim

**Affiliations:** ^1^ Department of Radiology, Chonnam National University Hospital, Gwangju, South Korea; ^2^ Department of Radiology, Chonnam National University Hwasun Hospital, Hwasun-gun, South Korea; ^3^ Department of Radiology, Chonnam National University, Gwangju, South Korea; ^4^ Department of Surgery, Chonnam National University Hwasun Hospital, Hwasun-gun, South Korea; ^5^ Department of Pathology, Chonnam National University Hwasun Hospital, Hwasun-gun, South Korea; ^6^ Department of Radiology, School of Medicine, Kyungpook National University, Kyungpook National University Chilgok Hospital, Daegu, South Korea; ^7^ Department of Artificial Intelligence Convergence, Chonnam National University, Gwangju, South Korea

**Keywords:** breast neoplasms, magnetic resonance imaging, machine learning, radiomics, ultrasonography

## Abstract

**Objective:**

This study was conducted in order to investigate the feasibility of using radiomics analysis (RA) with machine learning algorithms based on breast magnetic resonance (MR) images for discriminating malignant from benign MR-detected additional lesions in patients with primary breast cancer.

**Materials and Methods:**

One hundred seventy-four MR-detected additional lesions (benign, *n* = 86; malignancy, *n* = 88) from 158 patients with ipsilateral primary breast cancer from a tertiary medical center were included in this retrospective study. The entire data were randomly split to training (80%) and independent test sets (20%). In addition, 25 patients (benign, *n* = 21; malignancy, *n* = 15) from another tertiary medical center were included for the external test. Radiomics features that were extracted from three regions-of-interest (ROIs; intratumor, peritumor, combined) using fat-saturated T1-weighted images obtained by subtracting pre- from postcontrast images (SUB) and T2-weighted image (T2) were utilized to train the support vector machine for the binary classification. A decision tree method was utilized to build a classifier model using clinical imaging interpretation (CII) features assessed by radiologists. Area under the receiver operating characteristic curve (AUROC), accuracy, sensitivity, and specificity were used to compare the diagnostic performance.

**Results:**

The RA models trained using radiomics features from the intratumor-ROI showed comparable performance to the CII model (accuracy, AUROC: 73.3%, 69.6% for the SUB RA model; 70.0%, 75.1% for the T2 RA model; 73.3%, 72.0% for the CII model). The diagnostic performance increased when the radiomics and CII features were combined to build a fusion model. The fusion model that combines the CII features and radiomics features from multiparametric MRI data demonstrated the highest performance with an accuracy of 86.7% and an AUROC of 91.1%. The external test showed a similar pattern where the fusion models demonstrated higher levels of performance compared with the RA- or CII-only models. The accuracy and AUROC of the SUB+T2 RA+CII model in the external test were 80.6% and 91.4%, respectively.

**Conclusion:**

Our study demonstrated the feasibility of using RA with machine learning approach based on multiparametric MRI for quantitatively characterizing MR-detected additional lesions. The fusion model demonstrated an improved diagnostic performance over the models trained with either RA or CII alone.

## Introduction

Breast magnetic resonance imaging (MRI) is widely used for the preoperative evaluation of the extent of malignancy and the detection of any ipsilateral or contralateral additional lesions, especially for candidates for breast-conserving therapy (1). Magnetic resonance (MR)-detected additional lesions are the lesions found on preoperative MRI, which have not been identified on prior mammogram or ultrasound. Although predicting the malignancy of MR-detected additional lesions prior to surgery is of importance due to potential change in treatment strategies, it is a challenging task because of their small size and ambiguous imaging pattern. A second-look ultrasound (US) or a targeted US is generally performed to further evaluate the additional lesion (2, 3); however, US is usually unspecific for malignancy detection and the correlation rates between MR and US have been reported to be variable, ranging from 23% to 89%, depending on several factors, such as the performance of an individual radiologist or patient-specific differences (4). Although MR-guided interventions, such as a needle biopsy or a wire localization, may be performed, these procedures have a low priority because they are expensive, uncomfortable, and time-consuming compared with the second-look US. No defined protocol exists for the further workup of MR-detected additional lesion, whose clinical protocol usually relies upon the discretion of radiologists (2, 5).

Recently, there has been an increasing interest in developing a quantitative method of analyzing medical images. One such effort is the quantification of medical imaging data using radiomics analysis (RA). RA extracts a large number of quantitative features from medical images by applying pattern-characterizing mathematical formulas to gray-level pixel intensities that make up radiographical medical imaging data (6). The distinctive imaging features from RA have been shown to have the potential to reflect disease processes occurring at microscopic level (6). Breast MR images are the product of not only macroscopic breast tissue morphology but also abundant microscopic structures such as imperceptible tissue architecture, vascularization, and molecular diffusion. RA may be able to provide an insight on these microscopic environments and provide a non-invasive tool for the comprehensive understanding of the entirety of the tumor, which can add complementary information to the core-needle biopsy that assesses only a small portion of the tumor and, hence, present a potential localization error (7).

Using multiparametric breast MRI, RA has been applied to tumor characterization (8–10), detection of microcalcifications (11), prediction of response to neoadjuvant chemotherapy (NAC) (12, 13), classification of different molecular subtypes (14), and prediction of sentinel lymph node metastasis (15, 16) or cancer recurrence (17). In a recent study, Gibbs et al. demonstrated that RA based on high-resolution postcontrast images could differentiate between benign and malignant enhancing lesions that were subcentimeter in size (18). To our knowledge, no study has examined the use of RA based on multiparametric MRI for characterizing additionally detected lesions. Non-invasive prediction of malignancy from MR-detected additional lesions can facilitate a precise surgical planning by reducing time-consuming and costly procedures such as the second-look US and minimize unnecessary invasive procedures, including MR-guided biopsy.

The aim of this study was to investigate the utility of multiparametric preoperative MRI-based RA combined with machine learning algorithms in predicting the malignancy of additionally detected lesions in patients with primary breast cancer. Additionally, we developed a machine learning classifier trained using clinical imaging interpretation (CII) features that were generated by a qualitative MR imaging assessment by radiologists. We compared the performance of the RA classifiers to that of the CII classifier and investigated improvements with the RA+CII fusion model over their constituent models. Our model performances were validated using an external test set.

## Materials and Methods

### Patient Selection and Clinicopathologic Factor Evaluation

Institutional review board approval (CNUHH-2020-215) was obtained for this retrospective study, and informed consents from patients were waived.

**Figure 1:**
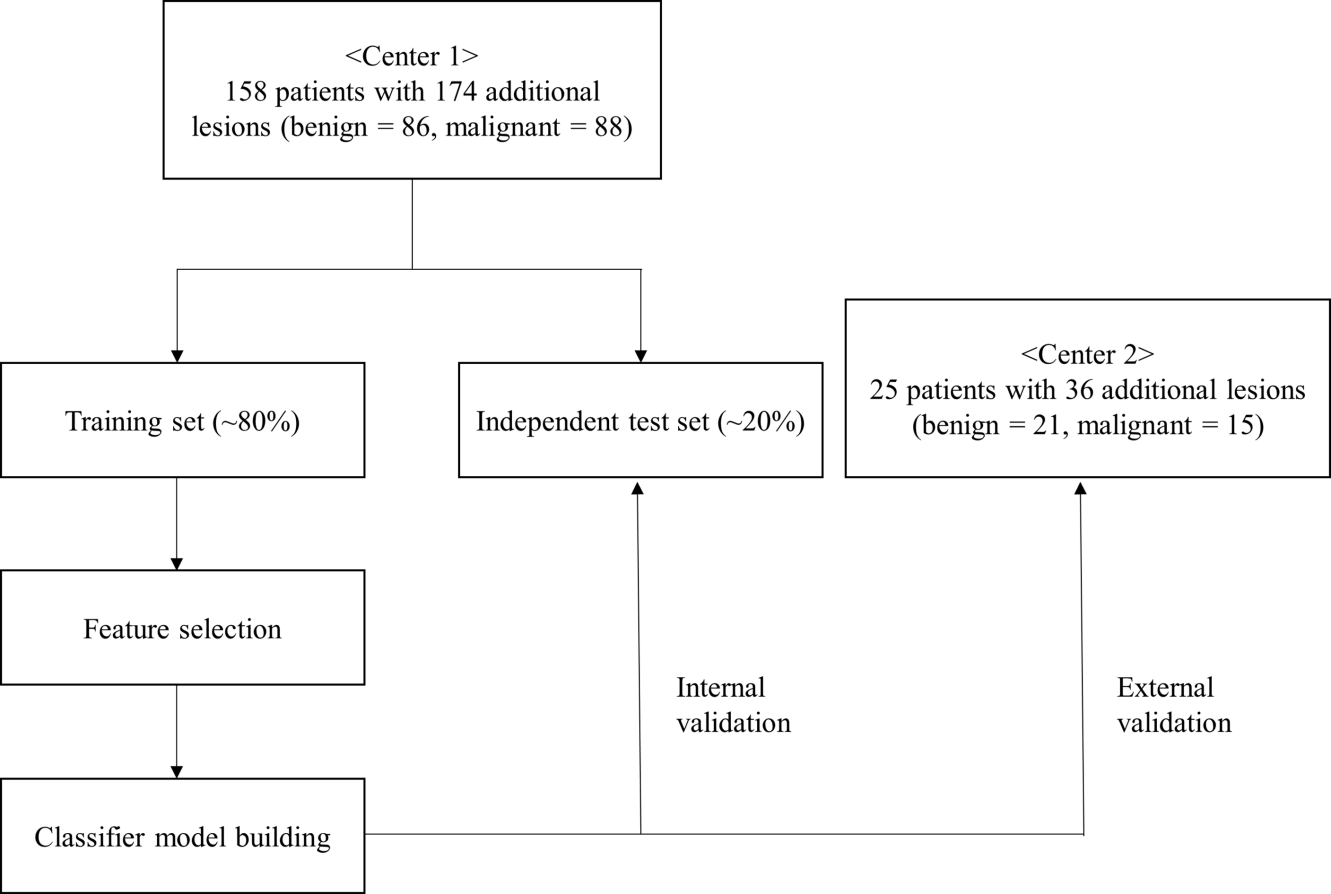
The workflow of the study.

Among a total of 6,558 breast MRI examinations that were performed at Chonnam National University Hwasun Hospital between January 2012 and July 2020, the MRI exams that met the following criteria were included: 1) initial breast MR images for preoperative evaluation of pathologically proven primary breast cancer; 2) interpretation reports containing the following keywords applied to ipsilateral breast lesion, “BI-RADS (Breast Imaging Reporting and Data System) 0,” “BI-RADS 4”, “BI-RADS 5”, “targeted”, “second-look”, or “US”; and 3) no history of NAC, excision, or vacuum-assisted biopsy. The following exclusion criteria were used: 1) additional lesions that were described as “daughter” or “satellite” lesions; 2) lesions that were interpreted as “non-mass enhancement” or “focus” according to the BI-RADS MR lexicon from the American College of Radiology (19); 3) lesions with size less than 7 mm, which were too small to extract sufficient radiomics features; 4) patients without a second-look US; 5) lesions that were sonographically occult or lacking a MR–US correlation; 6) lesions whose pathologic confirmation did not come from a separate excision; 7) lesions that were confirmed as a “borderline-risk” lesion, such as atypical ductal hyperplasia or lobular carcinoma *in situ* to avoid a doubtful ground truth of diagnoses; and 8) patients who were lost to follow-up. A total of 174 additional lesions (median size, 8 mm ranging from 7 to 15 mm; benign, *n* = 86; malignant, *n* = 88) were enrolled from 158 patients. The median age of the patients was 53 years (range, 25–73 years). For the external test set, 25 breast cancer patients with 36 MR-detected additional lesions (benign = 21, malignant = 15) from Kyungpook National University Chilgok Hospital, who met the same inclusion criteria, were enrolled to further validate our models. The schematic workflow of radiomics analysis is shown in **Figure 1**. All patients underwent mammography and US before MRI examination. All of the additional lesions were non-palpable and not detected on mammogram or US. A second-look US was performed after the MRI exam using a US platform with a 6–15-MHz linear probe (Logiq E9; GE Healthcare, Milwaukee, WI, USA). With reference to the preoperative MR images, lesion characteristics and depth as well as the location with regard to the surrounding tissue landmark were thoroughly considered using various techniques, including Doppler US and elastography, to ensure the MR–US correlation. A US-guided staining was performed for the suspicious lesion with a lower threshold for staining decision because MR-detected additional lesions often lack classical US findings for malignancy (20). The MR-detected additional lesions were histologically confirmed separately based on surgical operation performed on the same day of US-guided staining at our institution. The lesions that exhibited typical imaging findings for benign and, therefore, did not receive histology were considered benign if they remained stable on the follow-up imaging of at least 2 years.

Clinicopathologic factors including the age of the patient; histologic type, histologic grade, lymphovascular invasion (LVI), and Ki-67 (≥ 14% or <14% of the main tumor; histologic type of the additional lesion; pathologic tumor staging; immunohistochemical (IHC) subtype based on estrogen receptor (ER); progesterone receptor (PR); and human epidermal growth factor receptor 2 (HER2) positivity were investigated. Malignant pathologic diagnoses of the index tumor included ductal carcinoma *in situ* (DCIS), invasive carcinoma of no-special-type, invasive lobular carcinoma, mucinous carcinoma, papillary carcinoma, and medullary carcinoma. All the other pathologies without cancerous cells were considered benign. The automated IHC staining of ER, PR, HER2, and Ki-67 was performed for the pathological assessment of primary breast cancer. ER and PR positivity was scored by an Allred scoring system (21). HER2 staining scores were divided into four categories (0, 1+, 2+, or 3+) according to the ASCO/CAP guideline (22), with the score of 3+ considered as HER2-positive, the score of 0 or 1+ as HER2-negative, and the score of 2+ as equivocal, which needed a further assessment with *in situ* hybridization. Triple-negative breast cancer (TNBC) was defined as being ER- and PR-negative with the HER2 score of 0 or 1+ or with the absence of gene amplification.

### MRI Protocol

MRI was performed with 3-T scanners (TIM Trio, Skyra, Skyra II; Siemens Healthcare, Erlangen, Germany) with a dedicated breast coil with a minimum of four channels. Images of bilateral breasts were acquired in a prone position. The imaging parameters and protocols for the three types of scanners are presented in **Supplemental Table S1**. MRI exams consisted of axial fat-saturated turbo spin-echo T2-weighted imaging, dynamic contrast-enhanced imaging using T1-weighted axial 3D fat-saturated spoiled gradient-echo with the administration of gadoterate meglumine (Dotarem; Guerbet, Aulnay-sous-Bois, France) at a dose of 0.1 mmol/kg body weight, diffusion-weighted image (DWI), and apparent diffusion coefficient (ADC) map automatically generated by scanners. Dynamic contrast-enhanced MRI consisted of a precontrast and five postcontrast series with each series at a 60-s interval. Subtraction images were generated automatically from scanners by subtracting precontrast series from each of the five postcontrast series. The MRI data of the external test cohort were acquired using a 3-T scanner (Discovery MR750, GE Healthcare, Milwaukee, WI, USA).

### Clinical Imaging Interpretation of MRI Data

The CII features were obtained based on the BI-RADS MR lexicon by two board-certified breast radiologists with 3 and 16 years of experience, who were blinded to the clinicopathologic information. The CII features represent a mostly qualitative interpretation of radiographical imaging findings and consisted of morphology (mass or non-mass enhancement), size, shape, margin, and internal enhancement characteristics of the main tumor as well as size, shape, margin, internal enhancement characteristics, enhancement kinetic pattern of the additional lesion, and the additional lesion location with regard to the main tumor (same quadrant or different quadrant). In addition, background parenchymal enhancement (BPE) was also included. For 22 patients who possessed more than one additional lesion, the CII features were collected for each lesion.

For the analysis of enhancement kinetics for the additional lesion, a region-of-interest (ROI) was manually drawn using a postprocessing CAD system (CADstream, version 6.0; Confirma, Kirkland, WA, USA), after which early and delayed phase patterns were retrospectively analyzed according to BI-RADS descriptors. According to a previous study by Jansen et al. (23), the kinetic presentation of breast lesions may be inconsistent across different MR systems; therefore, we performed a qualitative assessment of kinetic curve shape. The early phase patterns consisted of slow (<50%), medium (50–100%), or rapid (>100%) enhancement patterns. The delayed phase patterns consisted of persistent, plateau, and washout components. The persistent, plateau, and washout components represented the pixel signal intensity with >10% increase, <10% increase and <10% decrease, and >10% decrease in the last postcontrast image compared with the first postcontrast image, respectively.

### MRI Intensity Normalization

Two sequences were selected from the whole breast MRI series on picture archiving and communicating system (PACS): the images obtained by subtracting pre- from the first postcontrast image (SUB) and T2-weighted image (T2). We used the SUB instead of the first postcontrast T1-weighted images because of its higher imaging contrast on a small lesion boundary compared with the latter. The SUB and T2 images were saved as a DICOM format. The simple ITK library (version 2.1.0; https://simpleitk.org/) was applied in Python to process the DICOM pixel values (24). In order to minimize inherent differences in pixel intensities across three different MR scanners, a *z*-score normalization was applied to the whole image voxels in every MR image (25). After the *z*-score normalization, the absolute value of the minimum pixel intensity was added to all pixels to convert negative pixel values to positive. The *z*-score algorithm was implemented from scratch using in-house written scripts in Python (http://www.python.org).

### Region-of-Interest Segmentation and Feature Extraction

Intratumor-ROIs were semiautomatically drawn by a breast radiology specialist with 3 years of experience using an open-source software, 3D-slicer (http://www.slicer.org). The intratumor-ROIs typically spanned 5–10 MR slices (slice thickness, 1.5–2 mm). In addition, peritumor-ROIs representing a donut-like region with a 5-mm extension from the tumor boundary were automatically obtained by dilating the delineated intratumor-ROI contour using a built-in function in 3D-slicer. The 2D ROIs, which were drawn on multiple slices, were rendered into a 3D ROI contour with an isotropic voxel resolution using a built-in function in 3D-slicer. Some additional lesions were challenging to be delineated on T2 due to a relatively large slice thickness of T2 (2.4 mm). As a result, a total of 22 T2 lesions were omitted. The mean ± SDs of intratumor, peritumor, and intra- and peri-tumor combined ROI volume were 342.5 ± 355.9, 906.9 ± 538.3, and 1,249.5 ± 880.0 mm^3^, respectively. A total of 107 original radiomics features were extracted using a PyRadiomics module in 3D-slicer from intratumor, peritumor, and the combined regions. The original radiomics features consisted of 14 shape, 18 first-order, and 75 texture features.

### Interobserver Reproducibility

Another board-certified radiologist drew ROIs for a total of 50 randomly selected patients to assess an interobserver reproducibility. Interobserver reproducibility of the extracted radiomics features from the ROIs drawn by the two radiologists was estimated by an intraclass correlation coefficient (ICC) based on two-way mixed effects model and interpreted according to the following criteria: excellent (>0.9), good (0.75–0.90), moderate (0.50–0.75), or poor (<0.50) (26).

### Feature Selection and Classifier Model Training for Radiomics Analysis

The patients at Chonnam National University Hwasun Hospital were randomly split to training (80%) and independent test sets (20%). To reduce the feature dimensionality and avoid overfitting, a penalized logistic regression with a least absolute shrinkage and selection operator (LASSO) analysis was applied across the training set 100 times with a random selection of LASSO training (80% of training set) and LASSO validation (20% of training set). Only features that rendered non-zero weights more than 10 times were selected (27). This process produced a total of 16 to 39 features. Next, the second feature selection step was applied to define a new set of features, *S*, in order to choose a combination of features that would produce the maximal classification accuracy. Each of the feature from the first step was added to *S* and the classification accuracy was evaluated. The support vector machine (SVM) was used to train and evaluate the classifier model (henceforth called the RA model) using the training dataset with a four-fold cross-validation, where three parts of the training dataset were used for training and one part for validation. Scikit-learn Python library was utilized for implementing SVM and LASSO (28). The accuracy was calculated as an average from the four-fold validations. If adding a new feature increased the accuracy, the feature remained in *S*. Otherwise, it was removed from *s*. After the optimal set of radiomics features were determined for SUB and T2, the features in *S_SUB_* and *S_T2_* were extracted from the independent test set, transferred to the trained SUB RA and T2 RA model, respectively, for evaluating model performances using the independent, internal test set. Another RA model was built using the full feature sets combining SUB and T2 radiomics features (henceforth called the SUB+T2 RA model). The feature selection and training procedure for the SUB+T2 RA model followed the same steps as those of the SUB and T2 RA models.

### Classifier Model Training for CII Features and Fusion Model

**Figure 2:**
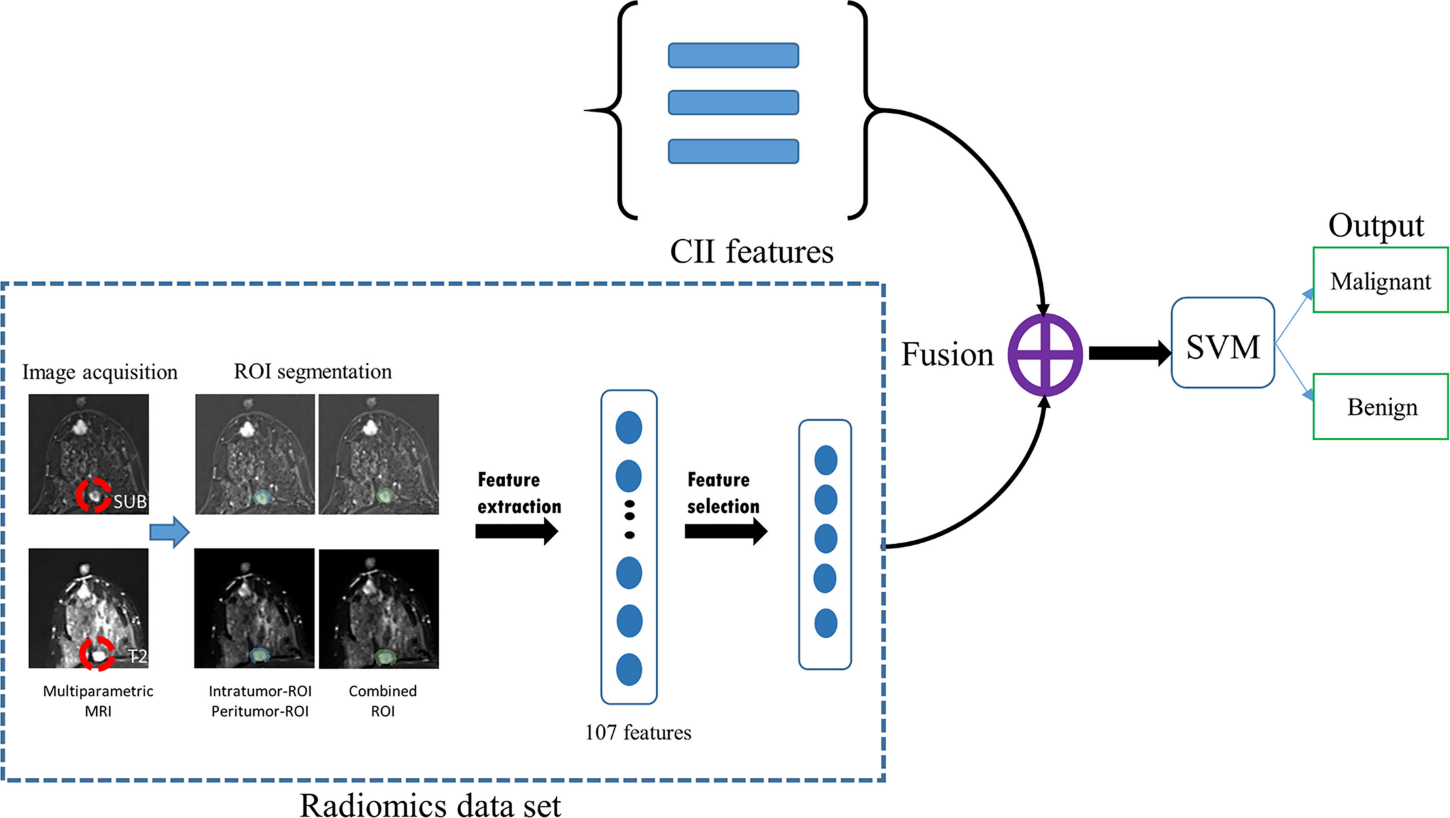
Schematic flowchart of the fusion model developed in this study. Radiomics features were extracted from MRI data using three regions-of-interest (ROIs; intratumor, peritumor, and combined). After the feature selection process, the selected radiomics features were concatenated with three CII features and used to train a support vector machine (SVM) for distinguishing between malignant and benign additional lesions. SUB, images obtained by subtracting pre- from the first postcontrast image; T2, T2-weighted image; MRI, magnetic resonance imaging, SVM, support vector machine; CII, clinical imaging interpretation.

A decision tree method was utilized to build a classifier model using the six CII features (henceforth called the CII model). These six features were selected after implementing univariate and multivariate analyses on the CII features. The names of these features are listed in **Supplemental Table S2**. The proposed decision tree model applied the ID3 algorithm to build up the tree (29). Lastly, fusion models combining radiomics and CII features were developed using SVM. The features in *S_SUB_* and *S_T2_* were separately concatenated with three CII features, namely, relative location to the main tumor, delayed kinetic pattern, and additional lesion margin, which were determined to be most attributable to the prediction in the CII model. After the prediction models were built, the performances of the models were further validated using the external test set. The schematic diagram for the fusion models is shown in **Figure 2**.

### Statistical Analysis

Clinicopathologic and CII features were compared between benign and malignant groups using Fisher’s exact test, chi-square test, or Mann–Whitney *U* test, where appropriate. Univariate and multivariate logistic regression analyses were used to evaluate a significant predicting factor for additional lesion malignancy.

To evaluate the RA and CII model performance, sensitivity (malignancy considered as a positive condition), specificity, accuracy with a threshold of 0.5, and the area under the receiver operating characteristic curve (AUROC) were calculated. The additional value of CII features to the RA classifier models was evaluated by net reclassification improvement (NRI) and integrated discrimination improvement (IDI) indexes. All statistical analyses were performed using SPSS software version 25.0 (SPSS Inc., Chicago, IL, USA) and R software version 3.5.1 (R Foundation for Statistical Computing, Vienna, Austria). All statistical tests were two-tailed with the *p*-value < 0.05 being considered significant.

## Results

### Patient Characteristics

The study population for the training and internal testing of the models included 79 patients with 82 benign additional lesions, 75 patients with 84 malignant additional lesions, and 4 patients with both benign and malignant additional lesions.

One hundred and fifty additional lesions were confirmed by histopathologic examination and the other 24 lesions were determined to be benign on imaging follow-up of at least 2 years. The histologic subtypes of the malignant additional lesions include DCIS (*n* = 7), invasive carcinoma of no-special-type (*n* = 62), invasive lobular carcinoma (*n* = 10), and others (*n* = 9), while histologic subtypes of the benign additional lesions include fibroadenoma (*n* = 33), adenosis (*n* = 4), fibrocystic change (*n* = 5), fibrosis (*n* = 7), benign intraductal papilloma (*n* = 8), and others (*n* = 5).

Clinicopathologic features between patients with benign and malignant additional lesions were compared. The patients with malignant lesions possessed a significantly higher number of axillary lymph node metastasis (*p* = 0.001) than those with benign lesions. The histologic type, histologic grade, LVI, Ki-67, pathologic T stage, and IHC subtype of the main tumor as well as the age of the patient were comparable between two groups (*p* > 0.05).

### Comparison of CII Features Between Benign and Malignant Additional Lesion

The comparison of the CII features between the benign and malignant lesions is shown in **Table 1**. The malignant lesion exhibited a significantly higher degree of minimal to mild BPE (*p* = 0.012) and higher degree of not circumscribed main tumor margin than the benign lesion (*p* = 0.006). All the other imaging features related to the main tumor (morphology, size, shape, internal enhancement) were similar between the two groups. In terms of imaging features related to the additional lesions, the malignant additional lesions showed a higher degree of irregular shape (*p* = 0.002), not circumscribed margin (*p* < 0.001), heterogeneous and rim enhancement (*p* = 0.007), and delayed washout kinetic pattern (*p* < 0.001) than the benign additional lesions. In addition, the malignant lesions were more likely to be located in the same quadrant as the main tumor (*p* < 0.001).

**Table 1:** Comparison of preoperative clinical imaging interpretation features between benign and malignant groups.

	Benign	Malignant	*p*
BPE			0.012
Minimal to mild	50 (61.0%)	67 (79.8%)	
Moderate to marked	32 (39.0%)	17 (20.2%)	
Main tumor morphology			0.131
Mass	75 (91.5%)	82 (97.6%)	
Non-mass enhancement	7 (8.5%)	2 (2.4%)	
Main tumor size (median, mm)	18.0	18.0	0.738
Main tumor shape			0.384
Round or oval	12 (16.0%)	9 (11.0%)	
Irregular	63 (84.0%)	73 (89.0%)	
Main tumor margin			0.006
Circumscribed	10 (13.3%)	1 (1.2%)	
Not circumscribed	65 (86.7%)	81 (98.8%)	
Main tumor internal enhancement			0.276
Homogeneous	2 (2.7%)	0	
Heterogeneous	51 (68.0%)	61 (74.4%)	
Rim enhancement	22 (29.3%)	21 (25.6%)	
Additional lesion size (median, mm)	8.0	8.0	0.828
Additional lesion shape			0.002
Round or oval	66 (76.7%)	48 (54.5%)	
Irregular	20 (23.3%)	40 (45.5%)	
Additional lesion margin			<0.001
Circumscribed	63 (73.3%)	34 (38.6%)	
Not circumscribed	23 (26.7%)	54 (61.4%)	
Additional lesion internal enhancement			0.007
Homogeneous	34 (39.5%)	19 (21.6%)	
Heterogeneous	46 (53.5%)	52 (59.1%)	
Rim enhancement	6 (7.0%)	17 (19.3%)	
Relative location to main tumor			<0.001
Same quadrant	32 (37.2%)	65 (73.9%)	
Different quadrant	54 (62.8%)	23 (26.1%)	
Early kinetic pattern^a^			0.052
Slow	0	0	
Medium	9 (11.1%)	7 (8.0%)	
Rapid	72 (88.9%)	80 (92.0%)	
Delayed kinetic pattern^a^			<0.001
Persistent	13 (16.1%)	1 (1.1%)	
Plateau	36 (44.4%)	14 (16.1%)	
Washout	32 (39.5%)	72 (82.8%)	

a Five benign additional lesions and one malignant additional lesion were not included for kinetics analysis due to motion artifact.

BPE, background parenchymal enhancement.

### Logistic Regression Analysis of Significant Clinicopathologic and CII Features

In univariate logistic regression, axillary lymph node metastasis (*p* = 0.001), BPE (*p* = 0.012), main tumor margin (*p* = 0.015), additional lesion shape (*p* = 0.002), additional lesion margin (*p* < 0.001), additional lesion internal enhancement (*p* = 0.013), relative location to the main tumor (*p* < 0.001), and delayed kinetic pattern of the additional lesion (*p* < 0.001) were significantly associated with malignancy of the additional lesion (**Table 2**). In multivariate analysis, additional lesion margin (*p* = 0.006), additional lesion internal enhancement (*p* = 0.045), relative location to the main tumor (*p* < 0.001), and delayed washout kinetic pattern of the additional lesion (*p* < 0.001) were significantly associated with malignancy of the additional lesion.

**Table 2:** Logistic regression analysis results.

Variables	Univariate analysis		Multivariate analysis	
	Odds ratio (95% CI)	*p*	Odds ratio (95% CI)	*p*
Axillary lymph node metastasis (yes/no)	3.354 (1.583–7.104)	0.001	2.614 (0.906–7.540)	0.075
BPE (minimal to mild/moderate to marked)	2.297 (1.198–4.405)	0.012	2.504 (0.972–6.446)	0.065
Main tumor margin (not circumscribed/circumscribed)	6.794 (1.456–31.699)	0.015	3.154 (0.505–19.707)	0.219
Additional lesion shape (irregular/round or oval)	2.750 (1.431–5.283)	0.002	2.253 (0.832–6.102)	0.110
Additional lesion margin (not circumscribed/circumscribed)	4.350 (2.289–8.267)	<0.001	3.431 (1.414–8.325)	0.006
Additional lesion internal enhancement		0.013		0.045
Homogeneous	Reference		Reference	
Heterogeneous	2.023 (1.017–4.022)	0.045	1.343 (0.477–3.786)	0.577
Rim enhancement	5.070 (1.710–15.034)	0.003	7.418 (1.505–36.558)	0.014
Relative location to main tumor (same/different quadrant)	4.769 (2.500–9.099)	<0.001	5.986 (2.493–14.376)	<0.001
Delayed kinetic pattern		<0.001		<0.001
Persistent	Reference		Reference	
Plateau	5.056 (0.603–42.354)	0.135	3.233 (0.326–32.060)	0.316
Washout	29.25 (3.668–233.23)	0.001	27.026 (2.834–257.72)	0.004

CI, confidence interval; BPE, background parenchymal enhancement.

### Interobserver Reproducibility

The ICC for all radiomics features showed excellent agreement. The ICCs (mean ± SD) of SUB intratumor-, SUB peritumor-, SUB combined-, T2 intratumor-, T2 peritumor-, and T2 combined-ROIs were 0.964 ± 0.06 (range, 0.580–0.999), 0.962 ± 0.04 (0.716–0.994), 0.988 ± 0.02 (0.814–0.999), 0.930 ± 0.11 (0.590–0.999), 0.957 ± 0.05 (0.636–0.996), and 0.974 ± 0.05 (0.642–0.999), respectively. The ICCs (mean ± SD) of features selected for the SUB, T2, and SUB+T2 RA models were 0.931 ± 0.09 (range, 0.795–0.998), 0.985 ± 0.02 (0.959–0.999), and 0.937 ± 0.10 (0.659–0.999), respectively. Therefore, the radiomics features from the first radiologist were used to train all RA models.

### Diagnostic Performance of RA and CII Models

The feature selection process selected five radiomics features from SUB to be used for training the SUB RA model. Similarly, five radiomics features from T2 were selected for training the T2 RA model. For the SUB+T2 RA model, a total of eight radiomics features were selected from the combined feature set from intratumor-SUB and intratumor-T2. The list of selected features for each model is provided in **Supplemental Table S2**. Among all RA models, two RA models based on the intratumor-ROIs of SUB and T2 yielded the highest classification performances with the testing accuracy and AUROC of 73.3% and 69.6% for SUB and 70.0% and 75.1% for T2, respectively (**Table 3**). The RA model based on multiparametric MRI data (SUB+T2 RA model) yielded a performance improvement over the RA models using single-modality MRI data with the accuracy and AUROC of 83.3% and 82.7%, respectively (**Table 4**). The CII model achieved a performance comparable to the RA models using intratumor-SUB and intratumor-T2 radiomics features. The accuracy and AUROC of the CII model were 73.3% and 72.0%, respectively (**Table 4**).

**Table 3:** Comparison of the RA model performances between various ROIs for differentiating malignant from benign MR-detected additional lesions.

Model		ROI	SUB				T2			
			Sensitivity	Specificity	ACC	AUROC	Sensitivity	Specificity	ACC	AUROC
**Training set (four-fold cross-validation)**										
RA	Intratumor		87.0 (81.1, 92.6)	58.0 (50.0, 66.2)	72.7 (65.3, 80.1)	79.4 (72.1, 86.8)	76.7 (69.2, 84.2)	69.4 (61.2, 77.6)	73.0 (65.1, 80.9)	74.4 (65.1, 80.9)
	Peritumor		64.3 (56.3, 72.3)	73.9 (66.6, 81.2)	69.1 (61.4, 76.8)	74.7 (61.4, 76.8)	79.0 (71.8, 86.2)	70.0 (62.0, 78.1)	74.6 (67.0, 82.3)	83.3 (67.0, 82.3)
	Combined		75.7 (68.6, 82.8)	60.9 (52.9, 69.1)	68.4 (60.7, 76.1)	73.5 (61.0, 76.0)	81.7 (75.2, 89.0)	56.5 (48.2, 65.8)	68.9 (61.0, 77.2)	74.6 (61.1, 77.0)
**Test set for internal validation**										
RA	Intratumor		93.5 (86.8, 100.0)	50.0 (42.5, 75.1)	73.3 (63.3, 91.1)	69.6 (50.0, 90.0)	73.3 (57.5, 89.1)	66.7 (50.0, 83.6)	70.0 (53.6, 86.4)	75.1 (53.1, 87.0)
	Peritumor		80.0 (65.7, 94.3)	47.7 (42.5, 65.6)	63.3 (46.1, 80.5)	68.0 (48.4, 87.6)	60.0 (42.5, 77.5)	60.0 (42.5, 77.5)	60.0 (42.5, 77.5)	66.0 (42.0, 78.1)
	Combined		73.3 (64.0, 91.6)	53.3 (42.0, 74.6)	68.6 (46.1, 80.5)	60.0 (38.3, 81.2)	73.3 (57.5, 89.1)	66.7 (50.0, 83.6)	70.0 (53.6, 86.4)	70.1 (53.1, 87.2)

Values are expressed as percentages, with 95% confidence intervals in parentheses.

ACC, accuracy; AUROC, area under the receiver operating characteristic curve; RA, radiomics analysis; ROI, region-of-interest; SUB, subtraction image; T2, T2-weighted image.

**Table 4:** Comparison of the performances between RA, CII, and RA+CII fusion models for the classification of malignant *vs*. benign MR-detected additional lesion.

Model	Training set				Internal test set				External test set			
	SEN	SPE	ACC	AUROC	SEN	SPE	ACC	AUROC	SEN	SPE	ACC	AUROC
SUB+T2 RA^a^	93.2 (88.4, 97.6)	83.6 (77.0, 90.2)	88.3 (82.5, 94.1)	91.8 (82.7, 94.1)	80.0 (65.7, 94.3)	86.7 (75.0, 99.0)	83.3 (70.0, 96.4)	82.7 (70.0, 97.1)	60.0 (44.0, 76.0)	85.7 (74.3, 97.1)	75.0 (60.9, 89.1)	88.6 (57.9, 87.8)
CII	87.0 (81.5, 92.6)	89.0 (83.8, 94.2)	88.2 (82.6, 93.4)	96.2 (82.5, 93.4)	66.7 (50.2, 83.8)	80.0 (65.7, 94.3)	73.3 (57.1, 89.0)	72.0 (57.1, 90.0)	73.3 (66.9, 93.1)	46.4 (30.1, 62.7)	66.7 (51.3, 82.1)	67.8 (51.9, 83.3)
SUB RA+CII^b^	86.0 (80.2, 92.0)	77.0 (70.0, 84.0)	81.2 (74.7, 87.7)	86.8 (73.0, 86.4)	66.7 (49.8, 83.5)	80.0 (65.7, 94.3)	73.3 (57.5, 89.1)	86.7 (57.1, 90.0)	80.0 (66.9, 93.1)	82.5 (70.1, 94.9)	83.3 (71.2, 95.5)	82.5 (69.9, 95.8)
T2 RA+CII^b^	80.0 (72.7, 87.3)	75.0 (67.1, 83.0)	77.6 (69.3, 84.7)	87.2 (70.0, 85.2)	86.7 (74.5, 99.0)	66.7 (50.0, 83.6)	76.7 (61.6, 92.0)	82.2 (61.4, 92.0)	93.3 (85.2, 99.9)	61.9 (46.0, 77.8)	75.0 (60.9, 89.1)	81.0 (65.1, 90.0)
SUB+T2 RA+CII^c^	85.5 (79.0, 92.0)	81.4 (74.3, 89.0)	88.3 (82.4, 94.2)	88.1 (76.5, 90.3)	86.7 (75.0, 99.0)	86.7 (75.0, 99.0)	86.7 (75.0, 99.0)	91.1 (74.1, 99.3)	80.0 (66.9, 93.1)	81.0 (79.5, 99.5)	80.6 (74.5, 97.3)	91.4 (66.9, 94.0)

Values are expressed as percentages, with 95% confidence intervals in parentheses.

RA, radiomics analysis; CII, clinical imaging interpretation; SEN, sensitivity; SPE, specificity; ACC, accuracy; AUROC, area under the receiver operating characteristic curve; SUB, subtraction image; T2, T2-weighted image.

a This model was trained using a combined feature set from intratumor-SUB and intratumor-T2.

b These fusion models were trained using a combination of CII features and radiomics feature from intratumor-SUB or intratumor-T2.

c This fusion model was trained using the radiomics features from the SUB+T2 RA model and CII features.

### Diagnostic Performance of RA+CII Fusion Models

**Figure 3:**
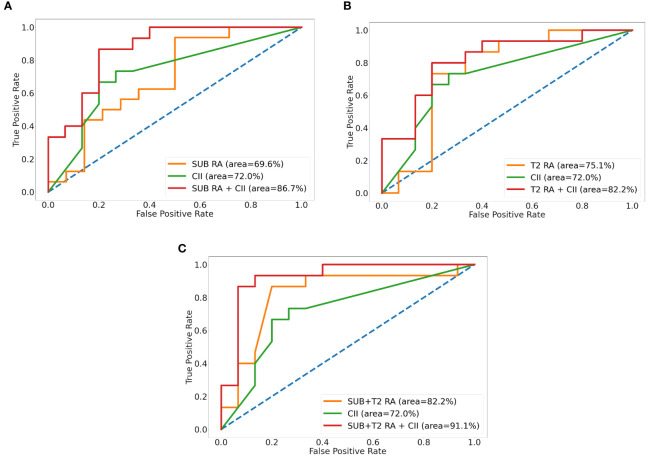
Receiver operative characteristic curves of radiomics analysis (RA), clinical imaging interpretation (CII), and fusion models for SUB **(A)**, T2 **(B)**, and SUB+T2 **(C)**. SUB, images obtained by subtracting pre- from the first postcontrast image; T2, T2-weighted image.

**Figure 4:**
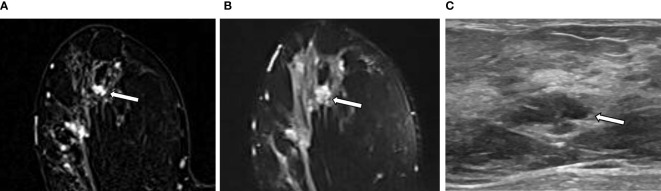
An example of a true negative result by the radiomics analysis (RA) model in a 36-year-old woman with invasive carcinoma of no-special-type in the right breast. **(A, B)** A 1.1-cm irregular heterogeneously enhancing mass (arrow) with high signal intensity on T2-weighted image is seen in addition to the index tumor [**(A)** axial first postcontrast T1-weighted image with subtraction; **(B)** axial T2-weighted image]. **(C)** Ultrasound image shows the corresponding 1.1-cm-sized mass with microlobulated margin (arrow). It was classified as suspicious lesion and the mass was excised. The RA model developed in this study categorized it as benign. The final histologic analysis revealed papilloma with epithelial hyperplasia.

**Figure 5:**
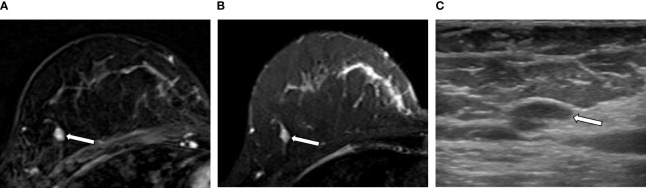
An example of a false negative result by the radiomics analysis (RA) model in a 49-year-old woman with invasive carcinoma of no-special-type in the right breast. **(A, B)** A 0.7-cm oval circumscribed homogeneously enhancing mass (arrow) with slightly high signal intensity on T2-weighted image is seen in the different quadrant of the same breast [**(A)** axial first postcontrast T1-weighted image with subtraction; **(B)** axial T2-weighted image]. **(C)** Ultrasound image shows the corresponding oval mass with 0.7 cm in size (arrow). The RA model developed in this study categorized it as benign; however, the final histologic analysis revealed ductal carcinoma *in situ*.

The intratumor-SUB and intratumor-T2 features from the RA models were concatenated with the three most discriminative CII features from the CII model to train fusion models. The performances of the fusion models were compared to those of the RA models and the CII model (**Table 4**). The fusion models combining the radiomics and CII features demonstrated improvements over the RA and CII models. The testing accuracy and AUROC of the SUB RA+CII model were 73.3% and 86.7%, respectively, which demonstrated a 14%–17% increase in AUROC compared with the AUROC of the SUB RA or CII models. Similarly, the testing accuracy and AUROC of the T2 RA+CII model were 76.7% and 82.2%, respectively, which indicated a 3%–7% increase in accuracy and a 7%–10% increase in AUROC compared with the corresponding metrics of the T2 RA or CII models. The fusion model (SUB+T2 RA+CII), which was trained with a combination of eight features from the SUB+T2 RA model and three features from the CII model, provided the highest performance with an accuracy of 86.7% and an AUROC of 91.1%, demonstrating a 3.4% and 8.4% increase in accuracy and AUROC, respectively, compared with the SUB+T2 RA model alone, and a 13.4% and 19.1% increase in accuracy and AUROC, respectively, compared with the CII model alone (**Table 4**). The ROC curves of RA, CII, and the fusion models are shown in **Figure 3**. In the testing result of the SUB+T2 RA+CII model, 2 out of 15 malignant lesions were misclassified as benign, and 2 out of 15 benign lesions were misclassified as malignant, rendering a sensitivity and specificity of 86.7%. Examples of true-negative and false-negative cases are illustrated in **Figures 4** and **5**, respectively.

The analysis of NRI and IDI between the RA and fusion models revealed that the addition of CII features significantly improved the performances of the SUB RA and T2 RA models. The NRI between the SUB RA and SUB RA+CII models was 0.80 (95% CI, 0.16–1.44; *p* = 0.014). The NRI between the T2 RA and T2 RA+CII models was 1.13 (95% CI, 0.57–1.70; *p* < 0.001). The addition of CII features to the SUB+T2 RA model, however, did not provide a significant improvement with the NRI between the SUB+T2 RA and SUB+T2 RA+CII models of 0.13 (95% CI, −0.58 to 0.85; *p* = 0.71). The IDI analysis demonstrated a similar pattern. The absolute IDI between the SUB RA and SUB RA+CII models was 0.32 (95% CI, 0.15–0.46; *p* < 0.001). The absolute IDI between the T2 RA and T2 RA+CII models was 0.16 (95% CI, 0.067–0.26; *p* < 0.001). The absolute IDI between the SUB+T2 RA and SUB+T2 RA+CII models was 0.015 (95% CI, −0.022 to 0.052; *p* = 0.43). Overall, these results suggest that the addition of CII features to the RA models enhanced the accuracy of malignancy prediction in the additional lesion with a significant improvement for the SUB and T2 RA models.

### External Validation

The external test cohort consisted of 19 patients with 20 benign additional lesions, 5 patients with 12 malignant additional lesions, and 1 patient with both 1 benign and 3 malignant additional lesions. The external test set was utilized to validate the performances of the SUB+T2 RA, CII, and the RA+CII fusion models (i.e., SUB RA+CII, T2 RA+CII, and SUB+T2 RA+CII). The results from the external test set are shown in **Table 4**. Overall, the results from the external test set were comparable to those from the internal test set. Similar to the result from the internal test set, the SUB+T2 RA+CII model, which was a fusion model between the SUB+T2 RA and CII models, provided the highest performance with an accuracy of 80.6% and an AUROC of 91.4%. In addition, the external test set exhibited the same pattern as the internal test set, in which the fusion models combining the radiomics and CII features demonstrated improvements over the RA and CII models. The accuracy and AUROC of the SUB+T2 RA+CII model with the external test set were 80.6% and 91.4%, respectively, which represented a 7%–20% increase in accuracy and 3%–35% increase in AUROC compared with those of the SUB+T2 RA or CII models.

## Discussion

This study demonstrated the feasibility of using RA with machine learning approach for predicting malignancy in MR-detected additional lesions. In both the internal and external test sets, the machine learning classifier that incorporated both radiomics and clinical MR imaging interpretation features provided high levels of accuracy (86.7% and 80.6% for the internal and external test sets, respectively) and AUROC (91.1% and 91.4% for the internal and external test sets, respectively), which indicated improvements over its constituent models that were trained using radiomics or clinical MR imaging interpretation features alone.

Previous studies of breast cancer have investigated the performance of RA based on breast MRI for the discrimination of benign and malignant lesions using radiomics features derived from multiparametric MRI data. Bickelhaupt et al. constructed a radiomics model based on T2-weighted image, DWI, DWI with suppression, and ADC map and reported that the radiomics model based on multiparametric MRI could differentiate between malignant and benign lesions with an improved performance compared with the model based on the ADC map alone (30). In another study by Bickelhaupt et al. (31), a machine learning model based on radiomics features from DWI and an adapted kurtosis fitting reduced false-positive cases, in which lesions were falsely classified as BI-RADS 4 or 5 at screening mammography. In a recent study, Zhang et al. reported that a machine learning model based on radiomics features from T2-weighted image, diffusion kurtosis imaging (DKI), and quantitative dynamic pharmacokinetic parameter map showed a reliable classification performance between benign and malignant breast lesions (32). Although these studies have shown the potential of using the combination of radiomics features from multiparametric MR modalities and machine learning algorithms for distinguishing malignant *versus* benign breast lesions, there has been no attempt to predict tissue characteristics of MR-detected additional lesions using the radiomics and machine learning approach based on multiparametric MRI.

It is of clinical significance to preoperatively identify tissue characteristics of MR-detected additional lesion because the additional lesions of patients with primary breast cancer have a high probability of being malignant and their identification can modify the original surgical plan and postoperative management strategy (33). There have been several efforts to evaluate the characteristics of MR-detected additional lesions. Nam et al. reported that the additional lesions with malignancy showed a significantly higher level of delayed washout kinetic pattern on dynamic contrast-enhanced MRI than those with benignity (20). Kim et al. reported that the probability of mass being malignant increased when lesions were located in the same quadrant as the main breast cancer (34). As in the cases of these efforts, the method to evaluate MR-detected additional lesions remains to be qualitative, with the BI-RADS categorization performed by a visual assessment. Although biopsy provides a central role in the diagnosis of breast lesion, its use is limited due to its invasive nature and it is often inadequate in providing a whole extent of tumor characteristics. On the contrary, medical imaging can provide a non-invasive way to repeatedly assess tissue characteristics of tumor and its surrounding and to evaluate their longitudinal changes throughout the whole disease management process (35). RA has been suggested as a new way to analyze medical imaging data and overcome the shortcoming of the conventional method of medical imaging analysis, which, for the most part, has relied upon a qualitative assessment of imaging findings. In this study, we attempted to develop a potential decision-assisting tool utilizing machine learning models based on the RA of multiparametric breast MR data for predicting malignancy of MR-detected additional lesions.

In the analysis of breast cancer, contrast-enhanced MR sequence is preferentially used because it provides information regarding the change in blood flow, which is a basis for differentiating cancerous from normal tissue. Interestingly, our results indicated that the RA model based on T2-weighted images showed a comparable performance to that based on SUB images, which were derived from contrast-enhanced MR sequences. Several previous studies have highlighted the usefulness of using T2-weighted image for breast cancer analysis. Zhang et al. demonstrated that a radiomics model based on T2-weighted images provided the second highest performance among the five models using various single-modality MR data (T2-weighted image, T1-weighted image, DKI, perfusion, ADC map), and a model based on the combination of T2, DKI, and perfusion imaging data provided the highest overall performance for discriminating between malignant and benign breast lesions (32). Parikh et al. pointed out that the changes in radiomics features derived from T2-weighted images were more sensitive to assess tumor heterogeneity after NAC than those from T1-weighted images (36). The authors have pointed out that the changes in vascularity during the course of therapy had possibly altered the degree of contrast enhancement.

In our study, the RA models based on the intratumor-ROI from both SUB and T2 sequences provided higher performances compared with those based on the peritumor- or combined ROI. For invasive breast cancer, the peritumoral region that includes the microenvironment beyond contrast enhancement or hyperintensity is believed to possess critical biological changes such as LVI, lymphocytic infiltration, or peritumoral edema (37, 38). Previous studies have shown that the peritumoral region was associated with chemotherapy response (39), sentinel lymph node metastasis (15), and patient outcome, including recurrence and survival (40). Although several studies have demonstrated the usefulness of including the peritumoral region for the analysis of breast lesion in the combined machine learning and RA approach (41, 42), one study has reported a different finding. Zhou et al. compared the diagnostic performance of deep learning models for the discrimination of benign and malignant breast lesions, while taking into account different extents of peritumoral region for comparison (8). They concluded that the use of the smallest bounding box to define tumor boundary and, therefore, the inclusion of minimal amount of peritumoral tissue generated a higher accuracy than the use of bigger bounding boxes that encompassed a larger area of peritumoral tissue. It is challenging to explain the results from the RA models using the peritumoral data in our study for the following reasons. First, we defined the peritumoral region as the area with a 5-mm extension from the tumor boundary, which was dictated by the limitation of the software used to draw ROIs. The 5-mm distance was relatively large considering the size of additional lesions, whose average volume was 342.5±355.9 mm^3^. The peritumoral region may have included a considerable amount of adjacent normal tissues and, therefore, affected the classification performance. Second, the use of a uniform distance to define the peritumoral region may be inappropriate to reflect the relevant peritumoral environment because of varying shape and size of the additional lesions. Unfortunately, no criteria exist to define the extent of peritumoral region beyond the tumor boundary. Further research is required to address the definition of peritumoral region and assess its effect on the radiomics approach.

Among the radiomics features consisting of the machine learning models, except for one shape feature, second-order statistics or texture features and skewness were more associated with the SUB-RA model, while first-order statistics or histogram features were mostly associated with the T2-RA model. Second-order statistics are related to the distribution of neighboring pixels or voxels within a tumor (43). Just described the meaning of high skewness as asymmetric distribution and lower mean value in histogram which could represent tumor progression on dynamic contrast-enhanced MRI (44). On the other hand, first-order statistics, which describe the simpler measures from the histogram of individual pixel intensity of the tumor, are considered to be more important for providing the information about tissue component in T2-weighted image, which is in line with Ha et al. (45).

Interobserver reproducibility in this study was determined by calculating ICCs in terms of ROI segmentation and computation, and the ROI segmentation may be the part where the variability is most likely to be introduced in RA research (46). In order to generate more reproducible ROIs, we utilized the semiautomatic segmentation options of 3D-slicer, which has been shown to produce ROIs with a greater reproducibility compared with the manual ROI segmentation (47). In addition, the target lesions in this study were relatively small-sized masses; thus, the boundaries of lesions were relatively easily identified and the ROI delineation of the tumor was relatively robust. For maintaining computational reproducibility, the same methods of outlier control, signal intensity control, and bin width were used between the different readers. In addition, we used a standardized, open-source module, PyRadiomics, for radiomics feature extraction. These efforts may have contributed to enhance the reproducibility of our radiomics features. Lastly, Van Griethuysen et al. reported that, among different types of radiomics features, the first-order, Laplacian, Gaussian-filtered, and texture features showed a relatively higher reproducibility compared with the shape or wavelet features (48). We did not utilize wavelet features, but 14 shape, 18 first-order, and 75 texture features, which may have contributed to the overall high ICCs.

We observed that the fusion model, which combines the radiomics and CII features, showed a higher accuracy and AUROC than the RA or CII models alone. It suggests that the combination of a qualitative evaluation by human efforts and a quantitative assessment by RA provided the maximal achievement in characterizing MR-detected additional lesions. Similar trends have been reported where the incorporation of RA and the conventional human expert labeling achieved better performance than a quantitative imaging analysis alone (49–51). Furthermore, the fusion model incorporating the radiomics features from multiparametric MR sequences and CII features exhibited the best overall performance, which indicated that the addition of relevant data can improve the training performance of a machine learning classifier. These results warrant that further optimization of the RA models with enriched clinicopathologic data and multimodal imaging data could provide higher feasibility and be used as an efficient adjuvant tool to support radiologist interpretation and clinical decision.

Three different MRI scanners were used to obtain the MR data used in this study. Even though the three scanners were manufactured by the same vendor and used the same imaging protocol to obtain breast MRI, the ranges of pixel intensity from these scanners were strikingly varied. These machine-dependent variances in signal intensity may be one of the sources for the lack of reproducibility in radiomics analysis (52, 53); thus, the imaging pixel intensities were normalized performed to minimize innate differences in MR signal between the three scanners. This effort will be valuable when the method developed in this study is to be applied in clinical practice where different types of MRI scanners are used. Our results showed that a high level of classification performance can be achieved for MR-detected additional lesions even when MR data with inherent difference in signal amplitude are used for training and testing the RA-based machine learning classifier.

This study has several limitations. First, to investigate the impact of peritumoral tissue, we included only mass lesions that had a clear boundary. It may be inappropriate to apply the RA model developed in this study to other datasets that include non-mass enhancement or foci. In addition, the additional lesions included in this study were limited to the ipsilateral lesions; thus, the number of data was relatively small. A study with a larger number of dataset including non-mass enhancement, foci, and the contralateral MR-detected additional lesions is currently under investigation. Second, it was a retrospective study with a relatively small sample size. Lastly, although we performed the external validation test with patient data acquired from another tertiary medical center and showed that the results were comparable to those of internal testing, the number of external testing cohort was relatively small. In addition, these data were collected in a diagnostic case–control manner, which might differ from the natural prevalence in real clinical setting. Medical data are highly heterogeneous and the artificial intelligence algorithms are known to be vulnerable to an overfitting problem. Our models may produce a suboptimal result in other datasets. A multicenter prospective study incorporating a larger number of newly enrolled patients is currently being planned, which may be able to further validate our approach.

## Conclusion

We have demonstrated the feasibility of combined radiomics and machine learning approach for differentiating benign and malignant MR-detected additional lesions in patients with primary breast cancer. Combining CII features with the RA model improved the classification performance. To our knowledge, our study represents the first attempt to use RA and machine learning approach for the characterization of MR-detected additional lesions and suggests that this method can potentially benefit these patient populations by reducing unnecessary and invasive procedures and, therefore, minimizing the associated complications, which will allow progress toward a personalized, precision medicine.

## Data Availability Statement

The raw data supporting the conclusions of this article will be made available by the authors, without undue reservation.

## Ethics Statement

The studies involving human participants were reviewed and approved by the Institutional Review Board of Chonnam National University Hospital. The ethics committee waived the requirement of written informed consent for participation.

## Author Contributions

H-jL and JEL: study design. H-jL, HSL, and IP: study conduct. MHP, JSL, and HJK: data collection and clinical data support. L-ND, A-TN, and IP: data processing and interpretation using machine learning. JEL, L-ND, and A-TN: statistical analysis. H-jL, SYK, HSL, and HJK: MRI reading. H-jL and A-TN: drafting manuscript. All authors: revising and approving manuscript content.

## Funding

This study was supported by the Ministry of Education, Republic of Korea (2019R1I1A3A01059201), and the Korea Health Technology R&D Project through the Korea Health Industry Development Institute (KHIDI), funded by the Ministry of Health & Welfare, Republic of Korea (HR20C0021).

## Conflict of Interest

The authors declare that the research was conducted in the absence of any commercial or financial relationships that could be construed as a potential conflict of interest.

## Publisher’s Note

All claims expressed in this article are solely those of the authors and do not necessarily represent those of their affiliated organizations, or those of the publisher, the editors and the reviewers. Any product that may be evaluated in this article, or claim that may be made by its manufacturer, is not guaranteed or endorsed by the publisher.
