# Supplementary material for: Classification of MR-Detected Additional Lesions in Patients With Breast Cancer Using a Combination of Radiomics Analysis and Machine Learning
Source: Front Oncol. 2021 Dec 2;11:744460. doi: 10.3389/fonc.2021.744460 (PMC8679659; doi:10.3389/fonc.2021.744460)
Supplement: Supplementary file 1 [file Table_1.docx]

**Supplemental Table S1**. Summary of parameters and imaging protocols of the three MRI scanners.

|  | Scanner 1 | | Scanner 2 | | Scanner 3 | |
| --- | --- | --- | --- | --- | --- | --- |
| Unit | 3-T Timtrio (Siemens) | | 3-T Skyra (Siemens) | | 3-T Skyra II (Siemens) | |
| Number of coil channels | 4 | | 16 | | 18 | |
| Flip angle | 10 | | 10 | | 10 | |
| Parallel imaging | iPAT2 | | iPAT3 | | iPAT3 | |
| Contrast agent | Dotarem 0.1-mmol/kg | | Dotarem 0.1-mmol/kg | | Dotarem 0.1-mmol/kg | |
|  | T1 | T2 | T1 | T2 | T1 | T2 |
| TR/TE (ms) | 4.5/1.6 | 6270/75 | 4.5/1.7 | 3840/70 | 4.5/1.7 | 3840/70 |
| Slice thickness (mm) | 2 | 2 | 1.5 | 2 | 1.5 | 2 |
| Matrix size (mm$\times$mm^2^) | 448$\times$314 | 384$\times$288 | 448$\times$358 | 384$\times$286 | 448$\times$358 | 384$\times$269 |
| FOV (mm$\times$mm^2^) | 320$\times$320 | 340$\times$340 | 320$\times$320 | 340$\times$340 | 320$\times$320 | 340$\times$340 |
| DWI acquisition parameter |  |  |  |  |  |  |
| Type of sequence | EPI-based | | EPI-based | | EPI-based | |
| Orientation | Axial | | Axial | | Axial | |
| In plane resolution (mm$\times$mm^2^) | 2$\times$2 | | 2$\times$2 | | 2$\times$2 | |
| Slice thickness (mm) | 4 | | 4 | | 4 | |
| Number of b values | 2 | | 2 | | 2 | |
| Lowest b value (sec/mm^2^) | 0 (not exceed 50) | | 0 (not exceed 50) | | 0 (not exceed 50) | |
| High b value (sec/mm^2^) | 800 | | 800 | | 800 | |
| Fat saturation | SPAIR | | SPAIR | | SPAIR | |
| TE (ms) | Minimum possible | | Minimum possible | | Minimum possible | |
| TR (ms) | $\geq$5000 | | $\geq$5000 | | $\geq$5000 | |
| Postprocessing | ADC map | | ADC map | | ADC map | |

TR= repetition time, TE= echo time, FOV= field of view, T1= T1-weighted image, T2= T2-weighted image, DWI= diffusion-weighted image, EPI= echo planar imaging, SPAIR= spectrally adiabatic inversion recovery

**Supplemental Table S2**. Names of radiomics and clinical imaging interpretation features

| ***ROI*** | ***Selected Feature Name*** |
| --- | --- |
| **SUB** | |
| Intratumor | Shape Maximum 2D Diameter Column |
|  | GLRLM Run Length Non-Uniformity Normalized |
|  | GLDM Dependence Variance |
|  | First Order Skewness |
|  | GLRLM Short Run Low Gray Level Emphasis |
| Peritumor | First order Interquartile Range |
|  | First order Root Mean Squared |
|  | GLSZM Size Zone Non Uniformity |
|  | First Order Maximum |
|  | Shape Least Axis Length |
| Combined | NGTDM Coarseness |
|  | First order Median |
|  | NGTDM Busyness |
|  | GLDM Large Dependence Low Gray Level Emphasis |
|  | GLRLM Gray Level Non-Uniformity |
| **T2** | |
| Intratumor | First Order 90 Percentile |
|  | First Order Maximum |
|  | First Order Median |
|  | First Order Variance |
|  | Shape Maximum 2D Diameter Slice |
| Peritumor | Shape SurfaceArea |
|  | Shape Maximum 2D DiameterColumn |
|  | GLDM Dependence Non-Uniformity |
|  | Shape Maximum 2D DiameterSlice |
|  | First order Median |
| Combined | Shape Maximum 2D DiameterRow |
|  | First order 90Percentile |
|  | First order Kurtosis |
|  | First order Variance |
|  | First order Range |
| **SUB + T2 (Intratumor)** | |
|  | Shape Maximum 2D Diameter Column |
|  | GLRLM Run Length Non-Uniformity Normalized |
|  | GLDM Dependence Variance |
|  | First order Skewness |
|  | GLRLM Short Run Low Gray Level Emphasis |
|  | First order 90Percentile |
|  | First order Maximum |
|  | First order Median |
| CII (values) | Relative location to main tumor (same quadrant, different quadrant) |
|  | BPE (minimal to mild, moderate to marked) |
|  | Additional lesion shape (irregular, oval or round) |
|  | Additional lesion margin (not circumscribed, circumscribed) |
|  | Additional lesion internal enhancement (rim enhancement, heterogeneous, homogeneous) |
|  | Delayed kinetic pattern (washout, plateau, persistent) |

ROI= region of interest, GLRLM= gray-level run length matrix, GLDM= gray-level dependence matrix, GLSZM= gray-level size zone matrix, NGTDM= neighborhood gray-tone difference matrix, CII= clinical imaging interpretation, BPE= background parenchymal enhancement
